# Directional Secretory Response of Double Stranded RNA-Induced Thymic Stromal Lymphopoetin (TSLP) and CCL11/Eotaxin-1 in Human Asthmatic Airways

**DOI:** 10.1371/journal.pone.0115398

**Published:** 2014-12-29

**Authors:** Gustavo Nino, Shehlanoor Huseni, Geovanny F. Perez, Krishna Pancham, Humaira Mubeen, Aleeza Abbasi, Justin Wang, Stephen Eng, Anamaris M. Colberg-Poley, Dinesh K. Pillai, Mary C. Rose

**Affiliations:** 1 Division of Pulmonary and Sleep Medicine, Children's National Medical Center, Washington, DC, United States of America; 2 Department of Pediatrics, George Washington University School of Medicine and Health Sciences, Washington, DC, United States of America; 3 Department of Integrative Systems Biology, George Washington University, Washington, DC, United States of America; 4 Center for Genetic Research Medicine, Children's National Medical Center, Washington, DC, United States of America; 5 Department of Biochemistry and Molecular Medicine, George Washington University, Washington, DC, United States of America; Imperial College London, United Kingdom

## Abstract

**Background:**

Thymic stromal lymphoproetin (TSLP) is a cytokine secreted by the airway epithelium in response to respiratory viruses and it is known to promote allergic Th2 responses in asthma. This study investigated whether virally-induced secretion of TSLP is directional in nature (apical vs. basolateral) and/or if there are TSLP-mediated effects occurring at both sides of the bronchial epithelial barrier in the asthmatic state.

**Methods:**

Primary human bronchial epithelial cells (HBEC) from control (n = 3) and asthmatic (n = 3) donors were differentiated into polarized respiratory tract epithelium under air-liquid interface (ALI) conditions and treated apically with dsRNA (viral surrogate) or TSLP. Sub-epithelial effects of TSLP were examined in human airway smooth muscle cells (HASMC) from normal (n = 3) and asthmatic (n = 3) donors. Clinical experiments examined nasal airway secretions obtained from asthmatic children during naturally occurring rhinovirus-induced exacerbations (n = 20) vs. non-asthmatic uninfected controls (n = 20). Protein levels of TSLP, CCL11/eotaxin-1, CCL17/TARC, CCL22/MDC, TNF-α and CXCL8 were determined with a multiplex magnetic bead assay.

**Results:**

Our data demonstrate that: 1) Asthmatic HBEC exhibit an exaggerated apical, but not basal, secretion of TSLP after dsRNA exposure; 2) TSLP exposure induces unidirectional (apical) secretion of CCL11/eotaxin-1 in asthmatic HBEC and enhanced CCL11/eotaxin-1 secretion in asthmatic HASMC; 3) Rhinovirus-induced asthma exacerbations in children are associated with *in vivo* airway secretion of TSLP and CCL11/eotaxin-1.

**Conclusions:**

There are virally-induced TSLP-driven secretory immune responses at both sides of the bronchial epithelial barrier characterized by enhanced CCL11/eotaxin-1 secretion in asthmatic airways. These results suggest a new model of TSLP-mediated eosinophilic responses in the asthmatic airway during viral-induced exacerbations.

## Introduction

The conducting airway epithelium in humans is organized as a pseudostratified columnar structure with a functional polarity and well-defined apical and basolateral compartments [1], [2]. Using proteomic analysis we have recently identified that this respiratory epithelial polarity is responsible for the presence of directional (apical and basolateral) airway secretomes [1]. Directional secretion is essential to regulate the molecular interactions between environmental challenges (apical) and sub-epithelial structures (basolateral) [1], [2]. For instance, apical recognition of allergens and pathogens through innate receptors (i.e. toll-like receptors, TLRs) may determine the nature of the inflammatory response generated in the sub-epithelial basolateral compartment [3], [4]. Understanding the directional immune response of the bronchial epithelial barrier may provide valuable insights about the pathogenesis of various respiratory disorders, such as asthma, that are characterized by airway inflammation elicited by apical environmental challenges, especially viruses [5], [6].

Respiratory viruses, the most common apical environmental challenges in asthma [5], [6], modulate innate Th1 and Th2 immune responses in the airways via the release of epithelial-derived cytokines [7]. Indeed, there is evidence that double stranded (ds) RNA, a viral surrogate that activates innate pattern recognition receptors in human bronchial epithelial cells (HBEC) [7], promotes sub-epithelial Th2 immune responses through the secretion of the Th2 master cytokine thymic stromal lymphopoetin (TSLP) [8]. Given that TSLP's primary function is to prime the differentiation of naïve T lymphocytes into Th2 cells via activation of antigen presenting cells [9]–[13], this crucial molecule is now considered the potential missing link between innate antiviral epithelial immunity and the Th2 atopic immune response characteristic of asthma [12], [13]. The pro-asthmatic effects of TSLP in the human asthmatic condition have been recently highlighted by a recent clinical trial that demonstrated that the administration of an anti-TSLP monoclonal antibody completely ablate allergen-induced bronchoconstriction and airway eosinophilic responses in asthmatic subjects [14].

Notwithstanding the compelling evidence supporting the pivotal role of TSLP in regulating the balance of antiviral Th1/allergic Th2 responses in the airways [13], [15], we still do not know basic details about the secretory biology of this molecule in the human airways. For instance, it is unclear whether virally-induced secretion of TSLP is directional in nature in HBEC (apical vs. basolateral) and/or if there are TSLP-mediated Th2 effects occurring at both sides of the bronchial epithelial barrier. To address these fundamental questions, we first examined the hypothesis that dsRNA stimulates bilateral (apical and basolateral) secretion of TSLP in primary HBEC differentiated at air-liquid interface (ALI) to form a polarized, pseudostratified epithelium. We also studied the specific effect of apical TSLP in differentiated HBEC and the potential sub-epithelial immunomodulatory action of TSLP in human airway smooth muscle cells (HASMC) obtained from control and asthmatic subjects. Extended studies investigated whether TSLP induces CCL11/eotaxin-1 in asthmatic HBEC and HASMC given the strong link between TSLP and airway eosinophilia in asthmatics [14]. Lastly, we conducted a clinical study in asthmatic children to examine if rhinovirus-induced asthma exacerbations are in fact associated with *in vivo* airway secretion of TSLP and CCL11/eotaxin-1. Collectively, our findings suggest a potential TSLP-driven autocrine mechanism occurring at the apical surface of the asthmatic respiratory epithelia that may amplify atopic responses via unidirectional (apical) secretion of CCL11/eotaxin-1 in the lumen of the airways of asthmatic subjects during viral respiratory infections.

## Methods

### Human bronchial epithelial cell (HBEC) cultures and *in vitro* differentiation

**Figure 1:**
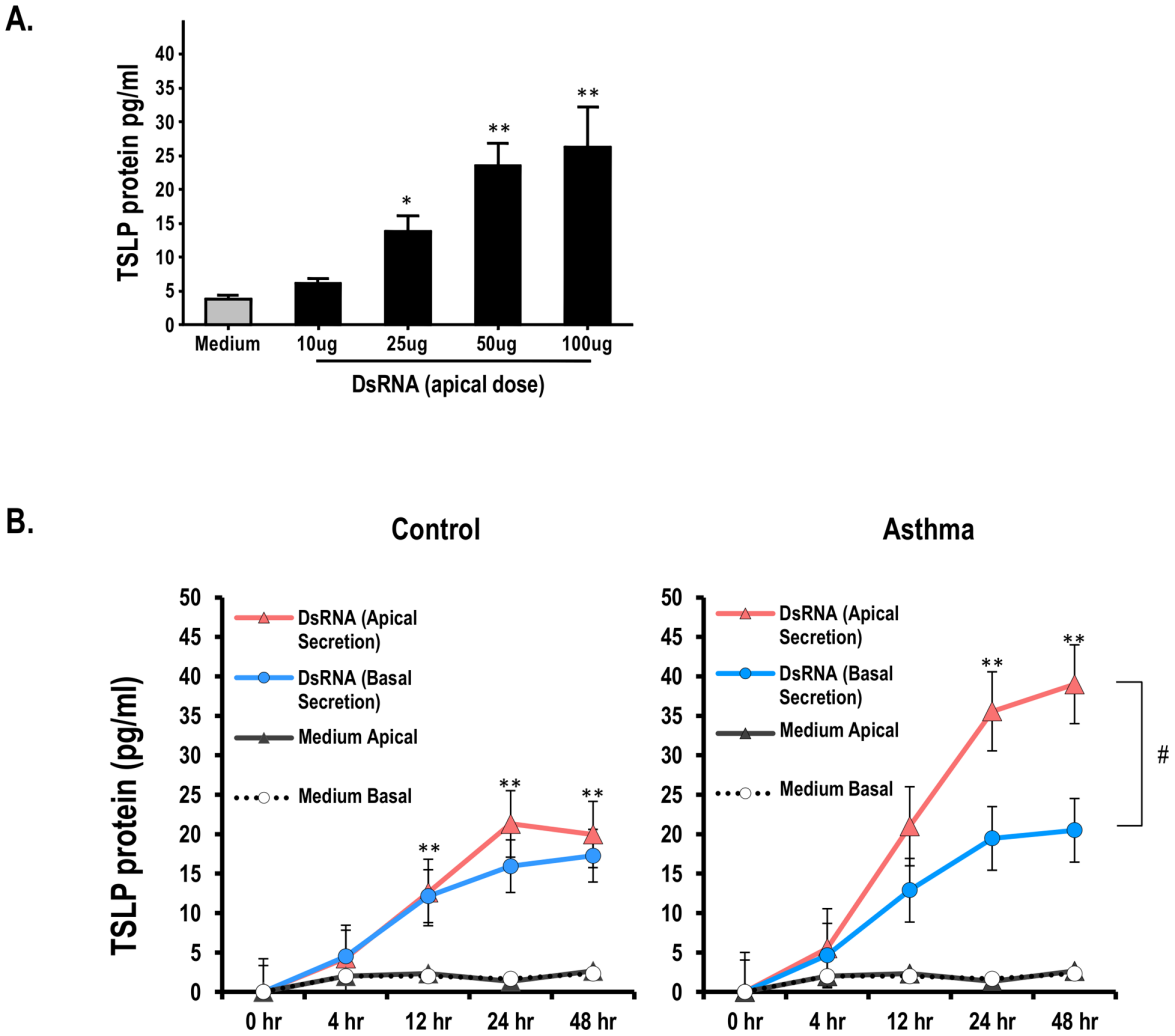
Double stranded (ds) RNA-induced secretion of thymic stromal lymphopoietin (TSLP) in human bronchial epithelial cells (HBEC). (A) Air-liquid interface (ALI) differentiated HBEC stimulated in the absence (medium) or presence of increasing concentrations (10, 25, 50, 100 ug/ml) of apical dsRNA (B) Time response of apical and basal TSLP secretion after apical administration of dsRNA (50 ug/ml) in ALI-differentiated HBEC. Data represent the means ± SE of triplicate values from 3 different asthmatic (n = 3) and control (n = 3) donors. **P<0.01 (DsRNA vs. medium), #P<0.05 (DsRNA-treated asthmatic HBEC apical vs. basal).

Human bronchial epithelial cells (HBEC) were purchased from Lonza, Walkersville, MD. Donors were three disease-free and non-smokers (Catalog number CC-2540, Lonza Inc., Switzerland) and three adult asthmatic subjects (Catalog number 194911, Lonza Inc., Switzerland). HBEC were amplified on collagen-coated T-75 flasks as previously described [1], [16], then plated apically on type IV collagen coated 12 well transwell plates (Fisher Scientific, Pittsburgh, PA), grown submerged for 7–10 days until 100% confluency. Apical media was removed and cells differentiated at air-liquid interface (ALI) to mimic a polarized conducting airway epithelium. After 20 days at ALI, cells were gently washed 4 times with PBS apically and baso-laterally and protein-free BEBM was added to the basal side. Confluent HBEC cells on ALI were stimulated apically under different experimental conditions including TSLP (10ng/ml) and dsRNA analogue (polyinosine-polycytidylic acid, poly I:C; InvivoGen San Diego, CA, USA). The poly I:C concentration used (50 ug/ml) was established by a dose-dependent experiment (Fig 1A). TSLP concentrations were selected based on prior studies [17]–[19]. Reagents remained in contact with HBEC for different time points as specified in the Figure Legends, after which HBEC supernatant, at the apical and basal secretions surface, was removed and maintained at −80°C until further analysis.

### Human airway smooth muscle cell (HASMC) culture and treatments

Human ASM cells (HASMC) were purchased from Lonza, Walkersville, MD. Donors were three adult asthmatic subjects (Catalog number 194850, Lonza Inc., Switzerland) and three disease-free and non-smokers (Catalog number CC-2576, Lonza Inc., Switzerland). HASMC were cultured in vitro as previously described [20], [21]. Briefly, HASMC were cultured in growth medium supplemented with 10% FBS (Bio Whittaker), and maintained in an incubator containing 5% CO2 in air at 37°C. After attaining ∼95% confluence, the cells were starved in unsupplemented Ham's F12 media for 24 hr. For all experiments cells were used at passage 2–4. HASMC were stimulated with either TSLP (10 ng/ml R&D Systems, Minneapolis, MN, USA) or dsRNA analogue (Poly I:C 10 ug/ml, polyinosine-polycytidylic acid; InvivoGen San Diego, CA, USA). Concentrations of these reagents were based on prior studies [21], [22]. After exposure to drugs and control media, confluent HASM cells were stimulated for 0, 24 or 48 h by adding dsRNA or TSLP to the wells. Cell supernatant was collected at the different time points and maintained at −80°C until further analysis.

### Nasal washing collection and viral PCR analysis

Nasal airway secretions were collected from hospitalized children aged 2–15 years seen in our medical center at the onset of their acute respiratory illnesses. Nasal washings were obtained by standard nasal lavage technique using saline (3–5 ml) and gentle suctioning of each nostril. For the *rhinovirus-induced asthma exacerbation group*, patients were eligible for the study if they had all of the following criteria: 1) history of physician-diagnosed asthma; 2) acute respiratory symptoms such as cough or shortness of breath; 3) documented wheezing; and 4) PCR (+) for rhinovirus. Subjects included in the *control group* were age-matched (2–15 years old), non-asthmatic children without clinical wheezing or detectable respiratory viral infection by PCR. Nasal samples were analyzed by a viral multiplex PCR panel for 14 targets used for clinical purposes in our institution (Luminex, TX, USA) according to the microbiology laboratory protocol. All clinical and demographic variables were obtained by reviewing electronic medical records at Children's National Medical Center. The Institutional Review Board (IRB) of Children's National Medical Center, Washington D.C. approved the study and granted a waiver of informed consent given that this research involved materials (data, documents, records, or specimens) collected solely for non-research purposes (clinical indications).

### Cytokine measurements

The supernatant from HASMC and HBEC (apical and basal) experiments and nasal washings were analyzed for protein levels of human TSLP and CCL11 (eotaxin-1). Cell supernatants were also assayed for CCL22 (macrophage-derived chemokine [MDC]), CCL17 (TARC), TNF-alpha and CXCL8 (IL-8). Cytokine levels were determined using a commercially available multiplex magnetic bead immunoassay (Millipore, MA, USA) according to the manufacturers' instructions.

### Statistical analysis

Data were analyzed using Minitab 16 software package for Windows (Minitab Inc., State College, PA, USA). All data are reported as mean +/− standard errors (SE), 95% confidence intervals (CI) or as fold changes relative to control values. Data within each group (asthma and control) and between asthma and control groups were analyzed with two sample t-test or non-parametric Mann Whitney U test when appropriate. For multiple comparisons one way ANOVA followed by post- test Bonferroni correction was used. Linear regression and Pearson correlation were used to evaluate the link between TSLP and CCL1/eotaxin-1 adjusting by covariates. A probability of <0.05 was considered statistically significant.

## Results

### DsRNA elicits a disproportionate apical secretion of TSLP in asthmatic Human Bronchial Epithelial Cells (HBEC)

Prior studies have reported that the TLR-3 agonist, dsRNA (poly I:C) induces TSLP secretion in submerged HBEC [8], [23]. However, the effect of dsRNA apically administered (viral infection mimic) to HBEC differentiated at air-liquid interface (ALI) is still unclear. An initial dose response study established that increasing concentrations of dsRNA increased TSLP secretion in a dose dependent manner and that 50 ug/ml was the optimal apical dsRNA dose to elicit appropriate TSLP secretion (apical) in ALI-differentiated HBEC (Fig 1A). We then exposed asthmatic (n = 3) and control (non-asthmatic, n = 3) HBEC to apical dsRNA (poly I:C, 50 ug/ml) and examined apical/basal TSLP secretion at different time points (0, 4 h, 12 h, 24 h and 48 h). As seen in Fig. 1B, there was a time-dependent increase in TSLP protein levels in both, the apical and basal compartments, with a maximal significant response at 24 h–48 h after treatment. There were no significant differences between the apical vs. basal TSLP peak responses in control HBEC (peak TSLP apical 21±SE 3.3 pg/ml vs. peak basal TSLP 18.8±SE 3.2 pg/ml, p>0.05, Fig 1B). In contrast, experiments conducted in HBEC obtained from asthmatic donors (n = 3) demonstrated that dsRNA elicited a greater TSLP response in the apical vs. basolateral compartment (peak TSLP apical 39±SE 3.2 pg/ml vs. peak basal TSLP 20.6±SE 3.5 pg/ml, p<0.05, Fig 1B). However, there were no significant differences in the basal secretion of TSLP observed in control vs. asthmatic HBEC after apical dsRNA exposure (Fig 1B).

### TSLP elicits apical secretion of CCL11/Eotaxin-1 in asthmatic HBEC

**Figure 2:**
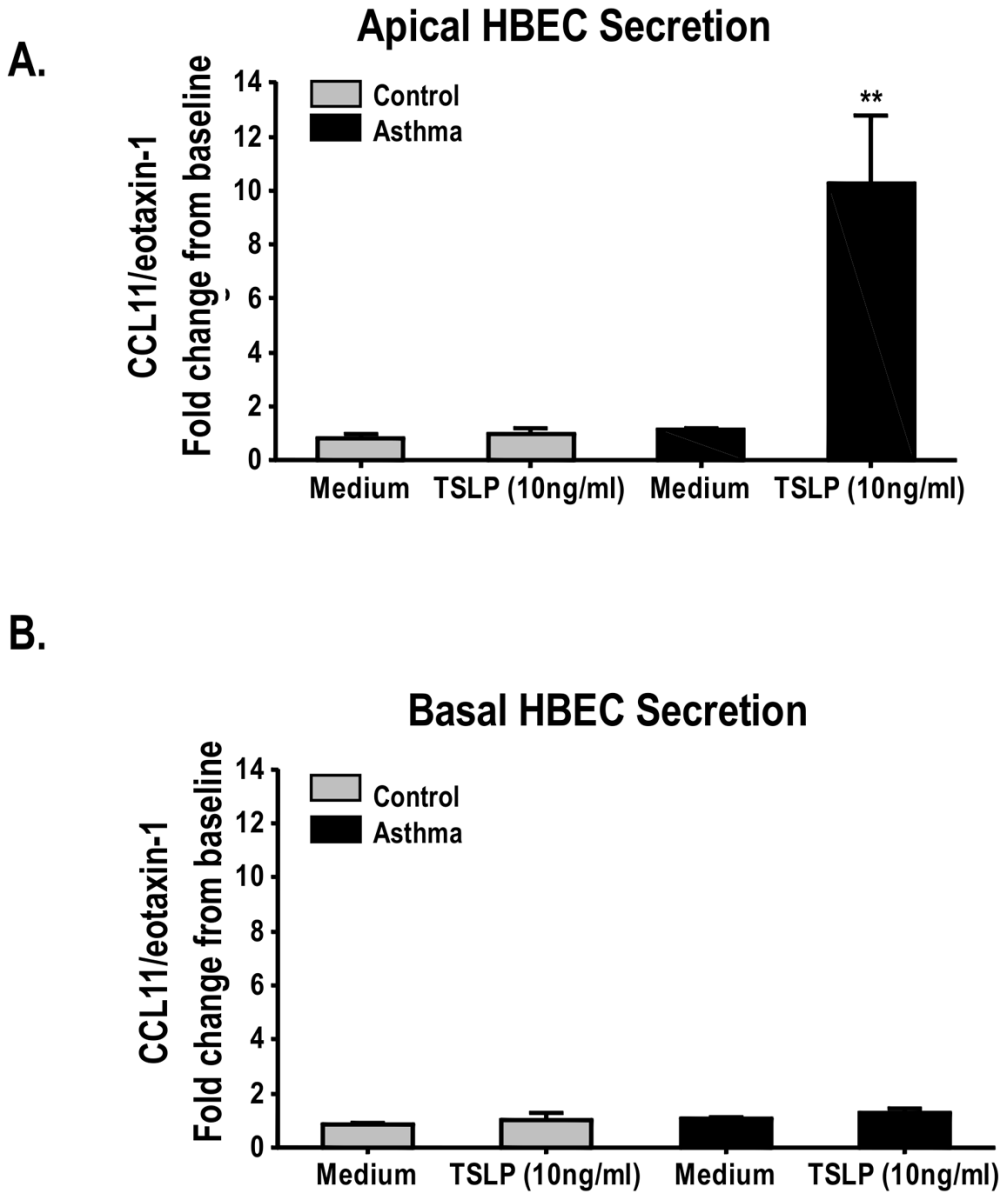
Unidirectional TSLP-induced secretion of CCL11/eotaxin-1 in HBEC. CCL11/eotaxin-1 is secreted apically (A) but not basally (B) by ALI-differentiated HBEC treated with TSLP (apical 10 ng/ml×48 h). Bars represent means ± SE of triplicate values from 3 experiments done with cells from 3 controls and 3 asthmatic donors. Results are expressed as fold increase from baseline relative to control values (medium alone). **P<0.01, *P<0.05.

There is increasing evidence linking TSLP to eosinophilic responses in the airways [14]. To investigate if the disproportionate secretion of TSLP in the apical side may potentially lead to secretion of the pro-eosinophilic C-C motif chemokine 11 (CCL11)/eotaxin-1, we exposed normal and asthmatic HBEC to apical TSLP (10 ug/ml during 48 h) based on previously reported optimal concentration [17], [19]. As shown in Fig. 2, asthmatic HBEC exhibited a unique polarized response to TSLP with significant apical, but not basal, secretion of CCL11/eotaxin-1 (fold increase 10.2±SE 2.5). Apical TSLP exposure did not elicit CCL11/eotaxin-1 secretion in HBEC from non-asthmatic donors (Fig 2). Of note, dsRNA did not elicit CCL11/eotaxin-1 secretion in HBEC from normal or asthmatic donors (data not shown).

### TSLP modulates the secretion of Th2-related chemokines and other pro-inflammatory molecules in HBEC

**Figure 3:**
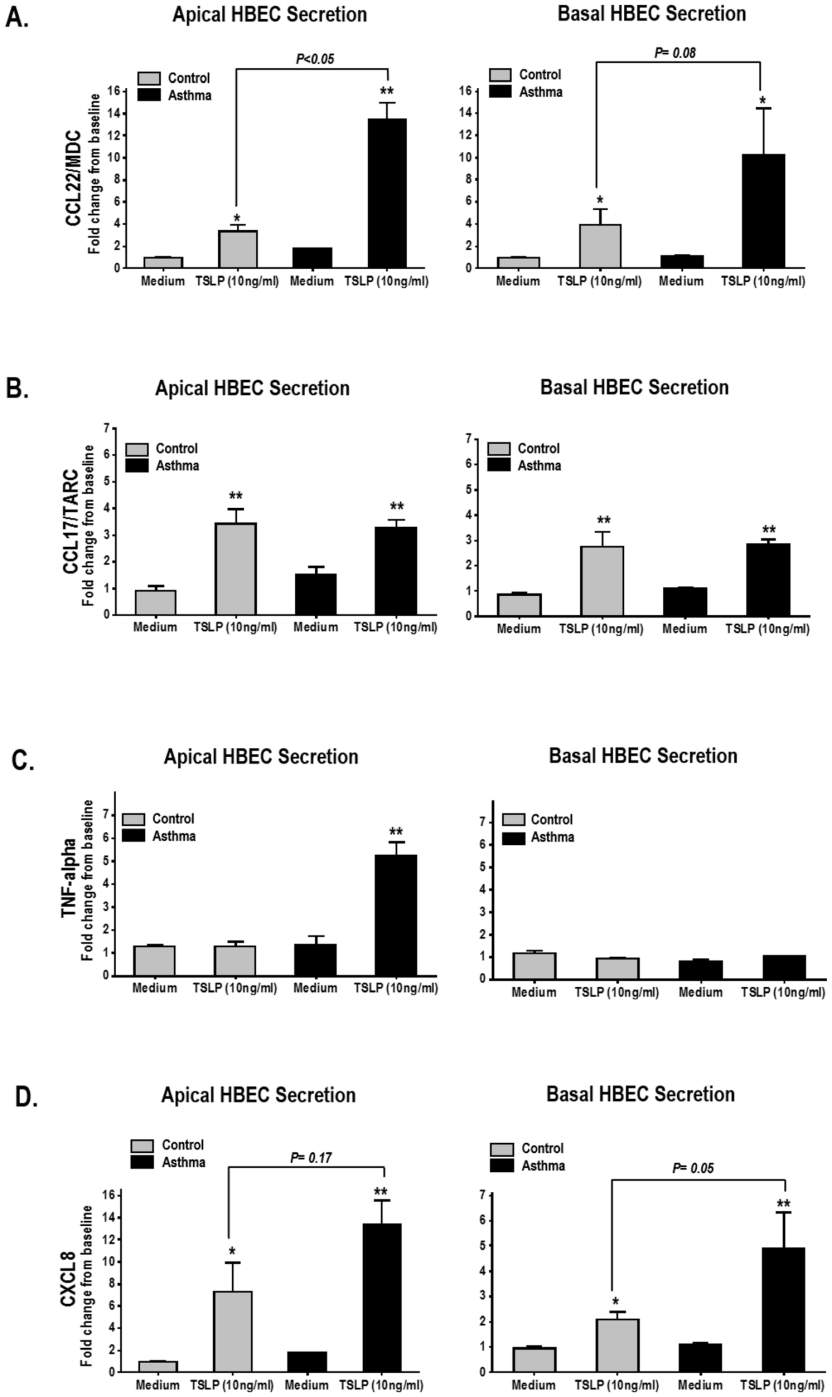
Bilateral TSLP-induced secretion of Th2-related chemokines and pro-inflammatory mediators in HBEC. CCL22/MDC (A),CCL17/TARC (B), TNF alpha (C) and CXCL8 (D) secreted by ALI-differentiated HBEC treated with TSLP (apical 10 ng/ml×48 h). Bars represent means ± SE of triplicate values from 3 experiments done with cells from 3 controls and 3 asthmatic donors. Results are expressed as fold increase from baseline relative to control values (medium alone). **P<0.01, *P<0.05.

We next investigated additional immune modulatory effects of the apical exposure of TSLP in HBEC. Fig. 3A-B illustrates that apical TSLP (10 ug/ml during 48 h) induced bilateral secretion (apical and basolateral) of two important Th2-related chemokines in asthma [13], C-C motif chemokine 22 (CCL22), also called macrophage derived chemokine (MDC), and the C-C motif ligand 17 (CCL17), well known as thymus and activation regulated chemokine (TARC). TSLP-induced CCL22/MDC secretion was more pronounced in apical asthmatic HBEC than in controls (Control HBEC: Baseline 83.5±SE 4.6 pg/ml and TSLP 48 h 270.5±SE 43.1 pg/ml, [fold increase 3.3±SE 0.6] vs. Asthmatic HBEC: Baseline 138.6±SE 49.4 pg/ml and TSLP 48 h 1745±SE 431 pg/ml, [fold increase 13.5±SE 1.4], p <0.01, Fig 3A). CCL17/TARC responses were comparable in control and asthmatic HBEC both, in the apical compartment (Control HBEC: Baseline 2.18±SE 0.6 pg/ml and TSLP 48 h 7.1±SE 1.2 pg/ml, [fold increase 4.9±SE 1.4] vs. Asthmatic HBEC: Baseline 2.04±SE 0.4 pg/ml and TSLP 48 h 7.3±SE 1.1 pg/ml, [fold increase 4.2±SE 0.9], p = 0.4, Fig 3B) and in the basal compartment (Control HBEC: Baseline 1.73±SE 0.4 pg/ml and TSLP 48 h 4.9±SE 0.9 pg/ml, [fold increase 3.2±SE 0.5] vs. Asthmatic HBEC: Baseline 1.5±SE 0.3 pg/ml and TSLP 48 h 4.0±SE 0.8 pg/ml, [fold increase 3.4±SE 0.2], p = 0.6, Fig 3B). In contrast, asthmatic HBEC exhibited a polarized response to TSLP with apical, but not basal, secretion of tumor necrosis factor (TNF) alpha (fold increase 5.2 ±SE 0.8, p <0.01, Fig 3C), which was not present in the basal secretion of asthmatic HBEC or in the apical/basal compartments of control HBEC. Apical exposure to TSLP also induced bilateral secretion of CXCL8 (IL-8), which was more prominent in the apical side of asthmatic HBEC (Fig 3D).

### Immune modulatory role of TSLP in Human Airway Smooth Muscle Cells (HASMC)

**Figure 4:**
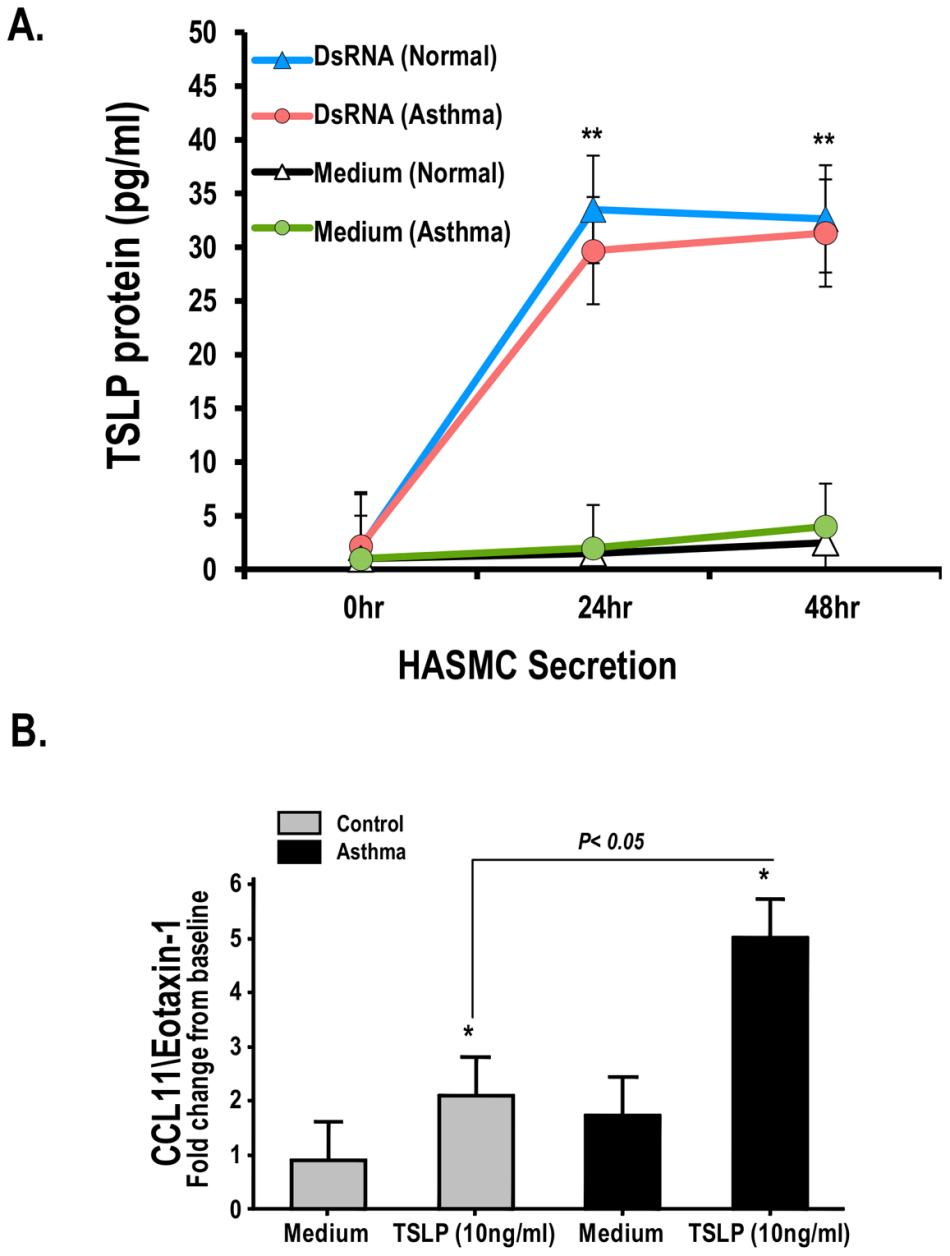
Antiviral and TSLP-induced immune responses in human airway smooth muscle cells (HASMC). (A) Time response of TSLP secretion after administration of dsRNA (10 ug/ml) in HASMC. (B) CCL11/eotaxin-1 secreted by HASMC treated with TSLP (10 ng/ml×48 h). Data represent the means ± SE of triplicate values from 3 different asthmatic (n = 3) and control (n = 3) donors. Bars data expressed as fold increase from baseline relative to control values (medium alone). **P<0.01, *P<0.05.

To examine the potential immune modulatory role of TSLP in the sub-epithelial compartment, we next evaluated the effect of TSLP exposure in HASMC. Initial experiments investigated whether dsRNA exposure elicits enhanced TSLP secretion in asthmatic HASMC as a potential source of sub-epithelial TSLP in the asthmatic state. As shown in Fig. 4A, there were time-dependent increments in TSLP protein levels secreted by HASMC exposed to dsRNA (poly I:C, 10 ug/ml). Maximal TSLP secretory responses were observed at 24 h–48 h in HASMC from healthy controls (n = 3) vs. HASMC from asthmatic donors (n = 3) (Fig 4A). Interestingly, there were no significant differences in the TSLP peak responses in control vs. asthmatic HASMC (peak TSLP 31±SE 4.1 pg/ml in control HASMC vs. peak TSLP 29±SE 3.5 pg/ml in asthmatic HASMC, p>0.05, Fig 4A). In contrast, HASMC exhibited increased secretion of CCL11/eotaxin-1 after TSLP exposure (10 ug/ml 48 h), which was more prominent in asthmatic vs. control HASMC (Fig 4B, fold increase 5±SE 0.7 in asthmatic HASMC vs. 2±SE 0.7 in control HASMC, p = <0.05). TSLP also induced the secretion of CXCL8 in control and asthmatic HASMC but it did not elicit the release of Th2-related chemokines (CCL22/MDC and CCL17/TARC) or TNF alpha in HASMC (data not shown).

### Airway Secretory Response of TSLP and CCL11/Eotaxin-1 in Rhinovirus-induced Asthma Exacerbations

**Figure 5:**
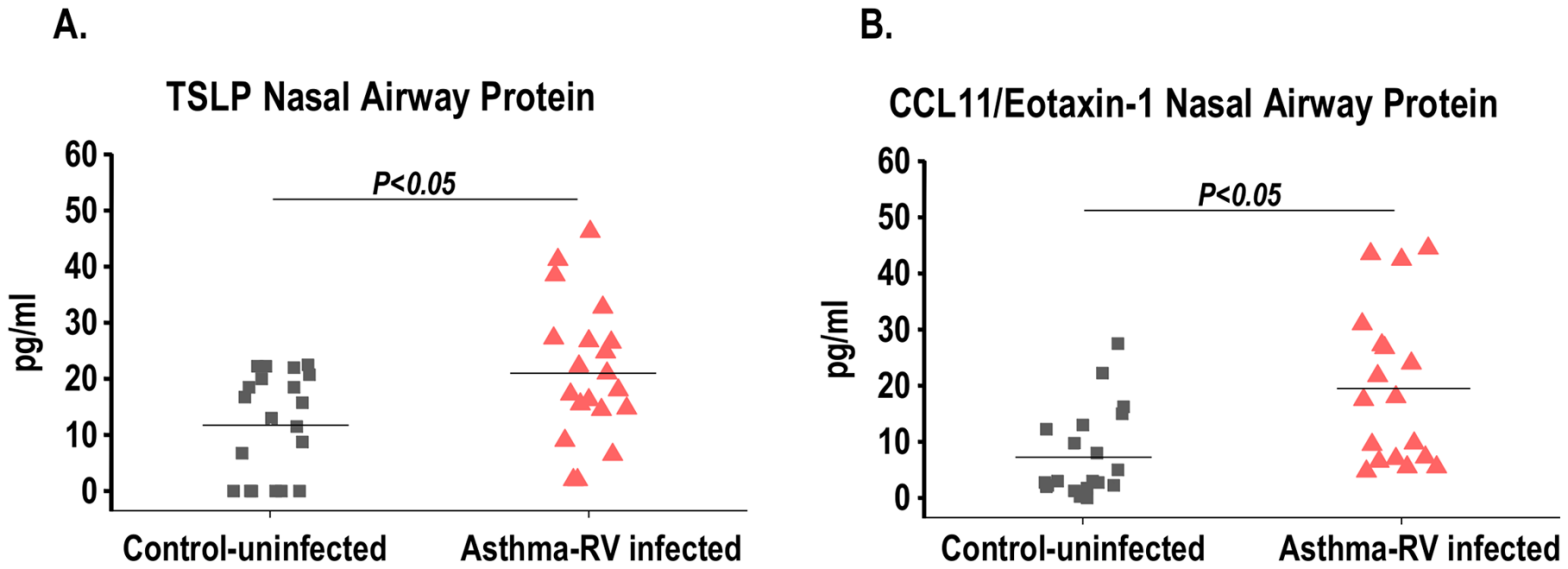
TSLP and CCL11/Eotaxin-1 secretion during rhinovirus-induced asthma exacerbation. Nasal airway protein levels of TSLP (**A**), CCL11/eotaxin-1 (**B**) in asthmatic children with PCR-confirmed rhinovirus and clinical wheezing (asthma; n = 20) vs. age-matched non-asthmatic children without clinical wheezing or rhinovirus (control; n = 20).

**Figure 6:**
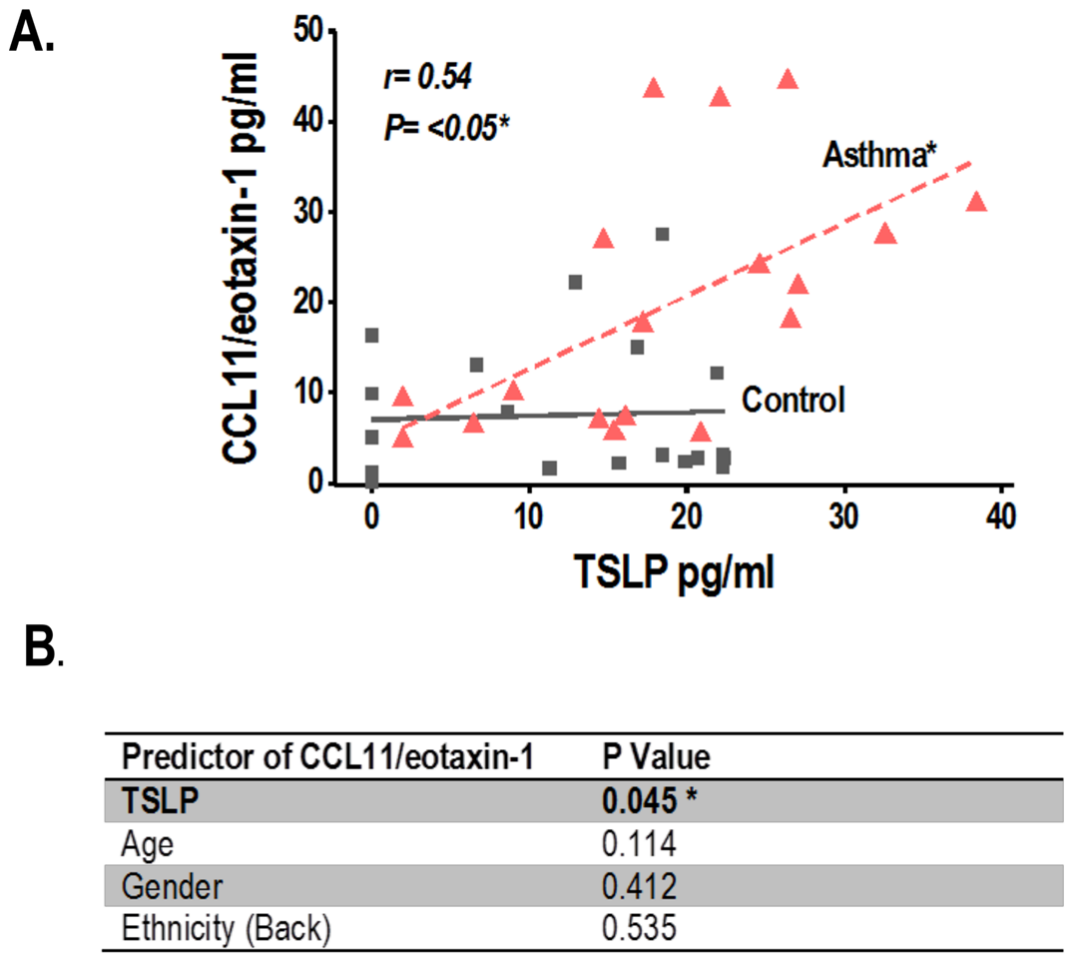
Linear correlation of TSLP and CCL11/Eotaxin-1 secretion during rhinovirus-induced asthma exacerbation. (A) Positive linear relationship of nasal airway protein levels of TSLP and CCL11/eotaxin-1 in subjects with rhinovirus-induced asthma exacerbation P<0.01 (r = Pearson correlation). (B) Multivariate linear analysis demonstrates that the link between TSLP and CCL11/eotaxin-1 in subjects with rhinovirus-induced asthma exacerbation (n = 20) is independent of age, gender and ethnicity. Values in boldface represent statistical significance (p<0.05).

To investigate if the TSLP and CCL11/eotaxin-1 responses of asthmatic airways *in vitro* pertain to the *in vivo* state, we conducted a clinical study contrasting TSLP and CCL11/eotaxin-1 nasal airway protein levels in children with rhinovirus-induced asthma exacerbation, defined as hospitalization of a known asthmatic child due to active wheezing and (+) rhinovirus PCR testing (n = 20) vs. age-matched, non-asthmatic controls without wheezing or detectable respiratory virus by PCR (n = 20). Table 1 shows that there were no significant differences in the baseline characteristics of enrolled subjects. Fig. 5 shows that relative to controls, asthmatic children with rhinovirus-induced exacerbation had significantly higher nasal protein levels of TSLP (20.9±SE 2.8 pg/ml in asthmatics and 11.9±SE 2 pg/ml in controls, p = 0.014, Fig 5A) and CCL11/eotaxin-1 (19±SE 3.8 pg/ml in asthmatics and 8.6±SE 2.2 pg/ml in controls, p = 0.026, Fig 5B). Moreover, TSLP levels correlated positively with elevated CCL11/eotaxin-1 levels in asthmatic children with rhinovirus-induced wheezing (Pearson correlation of CCL11/eotaxin-1 and TSLP = 0.54, p = 0.039, Fig 6A). Multivariate linear regression analysis demonstrated that the link between TSLP and CCL11/eotaxin-1 is independent of age, gender and ethnicity (adjusted p = 0.045, Fig 6B).

**Table 1:** Baseline characteristics for subjects.

Group	Control	Asthma
N	20	20
Male, n (%)	12(60)	11(55)
Age (y), mean (SD)	6.08 (5.1)	5.84 (4.7)
Black, n (%)	8(40)	12(60)

## Discussion

**Figure 7:**
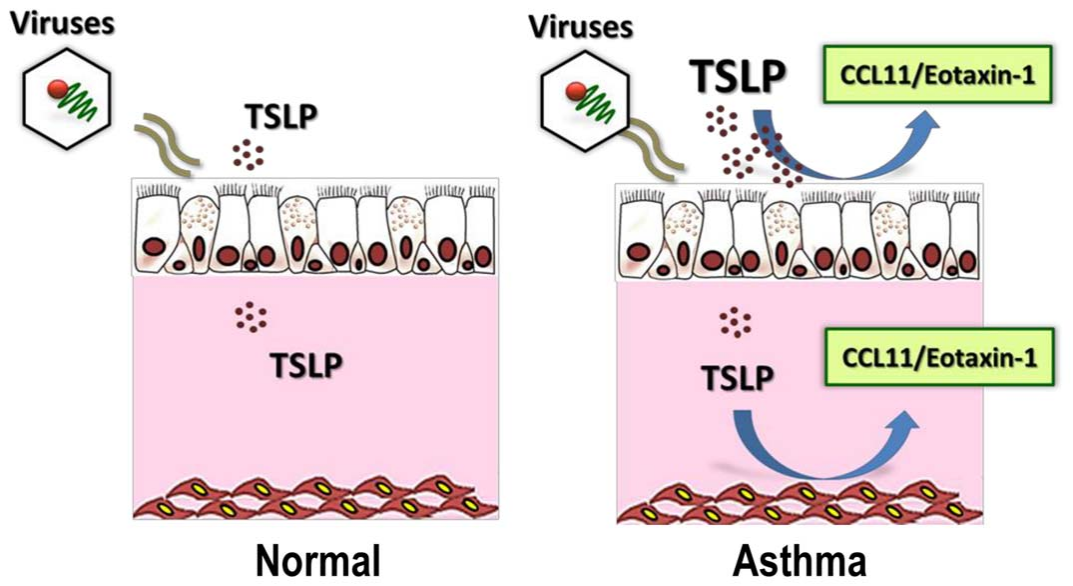
Model of TSLP-induced eosinophilic responses in the asthmatic airways during viral-induced exacerbations. After activation of airway antiviral immunity the asthmatic bronchial epithelium exhibit prominent secretion of TSLP in the apical compartment, which may amplify Th2 luminal responses (apical secretion) via secretion of CCL11/eotaxin-1. Sub-epithelial Th2 responses maybe further augmented by the TSLP-induced secretion of CCL11/eotaxin-1 by the underlying airway smooth muscle.

Thymic stromal lymphopoetin (TSLP), a master Th2 cytokine that promotes allergic responses in different cell systems [9]–[13], has been shown to be secreted from submerged (undifferentiated) human bronchial epithelial cells (HBEC) exposed to the double-stranded (ds) RNA (viral exposure mimic) [8] and in the nasal airway of children with rhinovirus [24]. TSLP has also been associated with the generation of airway eosinophilic responses during asthma exacerbations in human adult subjects [14]. Interestingly, the secretory biology of TSLP in asthmatic human airways is still largely unknown. In this study we examined the effects of TSLP at both sides (apical/basal) of the human epithelial barrier using a multi-scale approach that included an *in vitro* model of polarized, primary differentiated HBEC at air-liquid interface (ALI), human airway smooth muscle cells (HASMC) and clinical experiments in nasal airway secretions obtained during naturally occurring rhinovirus-induced asthma exacerbations. Our results identified that: 1) Apical dsRNA exposure induces bidirectional (apical/basolateral) secretion of TSLP in HBEC; 2) Asthmatic HBEC exhibit an exaggerated apical, but not basal, secretion of TSLP after dsRNA exposure; 3) TSLP exposure induces unidirectional (apical) secretion of CCL11/eotaxin-1 in asthmatic HBEC and enhanced CCL11/eotaxin-1 secretion in asthmatic HASMC; and 4) Rhinovirus-induced asthma exacerbations in children are associated with *in vivo* airway secretion of TSLP and CCL11/eotaxin-1. These data provide new insights about the nature of the TSLP-induced secretion of CCL11/eotaxin-1 at both sides of the bronchial epithelial barrier and suggest a new model of bilateral (apical and basolateral) TSLP-driven eosinophilic responses in the asthmatic airway during viral-induced exacerbations (Fig.7).

One the most important findings of our current study is that dsRNA induces a disproportionate amount of apical vs. basolateral TSLP secretion in ALI-differentiated asthmatic HBEC (Fig 1). DsRNA is an intermediate product of several RNA viruses (i.e. rhinovirus) that binds innate pattern recognition receptors such as toll-like receptor 3 (TLR-3), retinoic acid-inducible gene (RIG)-I, and melanoma differentiation-associated gene (MDA)-5, in a cell-type and pathogen-specific manner [25]. In the case of rhinovirus infection, the most common cause of asthma exacerbations at any age [26], dsRNA elicits TLR-3–mediated antiviral responses in the bronchial epithelium through the activation of TIR-domain-containing adapter-inducing interferon-β (TRIF) pathway [25], [27]. Importantly, submerged HBEC from asthmatic donors exhibit dysfunctional antiviral signaling in response to rhinovirus or dsRNA [25], [28], which results in overproduction of TSLP and attenuated secretion of type I interferons relative to control HBEC [23]. Similarly, dsRNA or rhinovirus infection enhances *in vivo* TSLP release in the airways of animal models of allergic asthma [13]. Using an *in vitro* model of a human asthmatic bronchial epithelium (ALI-differentiated HBEC), we now demonstrate that dsRNA exposure leads to time-dependent increases in unilateral (apical) TSLP secretion in asthmatic HBEC relative to control. DsRNA induces comparable levels of TSLP basolateral secretion in control and asthmatic ALI-differentiated HBEC. These data suggest that bilateral (apical/sub-epithelial) TSLP secretion is part of the normal innate antiviral immunity of the human bronchial epithelia, however, there is a distinct immune secretory signature in asthmatic individuals, consistent on dysfunctional apical TSLP hyper-secretion in response to dsRNA exposure. Future studies are necessary to confirm that our observations using dsRNA as a viral surrogate are also pertinent to infection with rhinovirus and other respiratory viruses.

TSLP is an epithelial-derived cytokine that contributes to the generation of allergic airway inflammation in asthma [10]–[13]. Epithelial and sub-mucosal bronchial samples from adult asthmatic patients contain enhanced TSLP mRNA expression [29], and bronchoalveolar lavage samples from these patients have higher concentrations of TSLP protein compared with those seen in healthy control subjects [29]. Furthermore, selective inhibition of TSLP prevents allergic airway inflammation in animal models of asthma [30] and completely ablates allergen-induced bronchoconstriction and airway eosinophilia in asthmatic subjects [14]. These later findings suggest a direct mechanistic link between TSLP and eosinophilic responses in the lung. In overall agreement with this notion, we identified that asthmatic HBEC exposed to TSLP leads to unidirectional (apical) secretion of CCL11/eotaxin-1 (Fig 2). CCL11/Eotaxin-1 is a Th2-related chemokine that activates the chemokine (C-C motif) receptor 3 (CCR3), a G protein coupled receptor that is essential for pulmonary eosinophil recruitment and the development of airway hyper-reactivity (AHR) in animal models [31]. CCL11/eotaxin-1 and CCR3 are co-localized in the bronchial mucosa of asthmatic patients and the intensity of their expression correlates with increased AHR [32]. Our current experiments in ALI-differentiated HBEC now suggest that TSLP-mediated induction of CCL11/eotaxin-1 in the apical compartment of the bronchial epithelia may lead to selective eosinophil accumulation in the lumen of the airways, a classical feature of the human asthmatic condition, particularly in severe cases [33].

Although TSLP and CCL11/eotaxin-1 play a critical role in the pathogenesis of asthma, it is noteworthy that TSLP alone was not enough to induce CCL11/eotaxin-1 in control HBEC. The latter indicates that there are intrinsic molecular differences between control and asthmatic HBEC that are collectively necessary for the development of the asthmatic condition. This concept is supported by genome-wide expression analysis of baseline and rhinovirus-infected HBEC from normal and asthmatic donors that have identified a unique transcriptomic pattern at baseline and during rhinovirus infection. [34] Complementing these genome-based observations, functional analysis of asthmatic HBEC have also identified dysregulated innate immune responses [3], [23] and abnormal repair mechanisms that promote airway remodeling [16], both of which are considered critical drivers of the asthmatic phenotype. Another important consideration in regard to the pro-asthmatic effect of TSLP in the airways is that in our airway epithelial model dsRNA did not elicit CCL11/eotaxin-1 secretion. The latter suggests that dsRNA-induced endogenous TSLP is not necessarily active under *in vitro* conditions. These findings are in agreement with the recent notion that there is a complex regulation of TSLP bioavailability in epithelial tissues. Indeed, TSLP protein is post-translationally activated by endogenous proteases in nasal polipoid tissue [35] and in the skin [36]. Notably, Briot et al. have recently reported that there is a biological cascade elicited by Kallikrein 5 (KLK5) that is crucial for the bioactivity of TSLP in the epidermis and so may play a role in the pathogenesis of atopic dermatitis [36]. Although the mechanisms that determine local activation of TSLP in the airways still need to be elucidated, our *in vitro* results suggest that there might be additional factors that determine TSLP activity in the *in vivo* microenvironment of the human airway epithelium.

Extended *in vitro* experiments identified that TSLP also induces unidirectional (apical) secretion of TNF-alpha in asthmatic HBEC (Fig 3). Since TNF-alpha heightens the development of airway TSLP/Th2 pro-asthmatic responses after dsRNA exposure [37], our findings suggests that TNF-alpha may amplify the potential effect of TSLP in viral-induced asthma exacerbations. Further supporting this concept is the recent evidence that TNF-alpha induces TSLP secretion in differentiated HBEC [28], [38], which has been previously described in different cell systems [39]. More studies are needed to investigate if the complex regulatory interplay between TNF-alpha and TSLP is dysregulated in the asthmatic condition, as suggested by our current results. In addition to CCL11/eotaixn-1 and TNF-alpha, HBEC exposed to TSLP induced the secretion of CCL22/MDC and CCL17/TARC, two CCR4-ligands that are considered critical Th2-related chemokines in asthma exacerbations [40]. CCL22/MDC and CCL17/TARC were secreted bilaterally (apical/basal) in control and asthmatic HBEC, although asthmatic donors showed higher CCL22/MDC apical responses (Fig 3). Collectively, these results suggest that virally-induced TSLP apical secretion modulate the immune secretory phenotype in asthma, characterized by the production of Th2-related chemokines in the bronchial epithelium during acute exacerbations [40].

Our study found that cytokines and chemokines are produced differentially in apical and basal sides of the human bronchial epithelium, which is in agreement with our prior proteomic analysis that identified directional (apical and basal) airway secretomes [1]. In this context, it is important to mention that although it is not fully understood why cytokine/chemokine production shows specific apical/basal patterns in the human bronchial epithelium, it is possible that these directional secretory patterns are due to intrinsic differences in the molecular phenotype and biology of the airway basal epithelial cells [41]. In support of this notion is the work of Hackett and colleagues [41] that recently identified the “human airway basal cell signature” consistent on 1,161 unique genes with >5-fold higher expression level in basal cells compared to differentiated epithelium. The physiological role of these differences is illustrated by the hierarchical mapping of Gene Ontology categories enriched in the human basal cell transcriptome [41] and basal vs. apical secretomes [1], which suggested clear differences in the predicted functional and cell-to-cell attributes of apical and basal compartments. Futures studies are needed to investigate if human airway basal cells exhibit differential secretion of cytokines/chemokines (i.e TSLP and CCL11) and if this immune responses are abnormally regulated in the asthmatic condition.

In concert with effects in the bronchial epithelium, TSLP also directly targets the sub-epithelial human airway smooth muscle cells (HASMC) via activation of the TSLP receptor-(R), which has constitutive and inducible expression in this airway cell type [42], [43]. Importantly, TSLP expression is increased in the bronchial airway smooth muscle bundles of asthmatics [42], [43], suggesting that TSLP may exert sub-epithelial autocrine/paracrine roles [43]. In our current work we identified that dsRNA elicit time-dependent secretion of TSLP in HASMC, but that this immune response is not different in asthmatic vs. control preparations (Fig 4A]. Given that TSLP-R activation in HASMC also elicits the release of pro-inflammatory mediators such as CCL11/eotaxin-1 [22], we compared immune secretory responsiveness to TSLP in control vs. asthmatic HASMC. Akin to the apical secretory response seen in asthmatic HBEC, asthmatic HASMC exhibited enhanced TSLP-induced secretion of CCL11/eotaxin-1 relative to controls (Fig 4B). These data indicated that asthmatic airways have a dysregulated hyper-responsiveness to TSLP that potentially leads to augmented CCL11/eotaxin-1 secretion at both sides of the bronchial epithelial barrier (Fig 7). In this model, the sub-epithelial TSLP/CCL11/eotaxin-1 responses of the asthmatic bronchial smooth muscle might underlie the presence of airway wall eosinophilc infilitration, one of the most striking features in the pathology of severe asthma [44].

To investigate the clinical relevance of the proposed model of TSLP-driven eosinophilic responses via CCL11/eotaxin-1 secretion in the asthmatic airways, we conducted a clinical study to see if virally-induced asthma exacerbations are associated with *in vivo* airway secretion of TSLP and CCL11/eotaxin-1. In this “proof of concept” cross-sectional study we collected nasal secretions of asthmatic children with acute rhinovirus-induced wheezing leading to hospitalization (viral-induced asthma exacerbation) and compared them with age-matched, non-asthmatic children without wheezing or detectable virus (control). We identified that TSLP and CCL11/eotaxin-1 levels were not only significantly elevated in the group with rhinovirus-induced asthma exacerbation (Fig 5), but that their levels had a linear correlation independently of age, gender and ethnicity (Fig 6). It is important to emphasize that in our study the control group only included non-asthmatic children without RV. Accordingly, based on our current findings we cannot conclude that RV-induced TSLP secretion is specifically present in asthmatic subjects. Longitudinal studies that include a control group of non-asthmatic subjects are still needed to clarify this important point. Nonetheless, our current results support the novel concept that there is apical (luminal) TSLP-induced secretion of CCL11/eotaxin-1 in the asthmatic airways. CCL11/eotaxin-1 could play a key role in mediating the pro-asthmatic effects of TSLP in the airways of asthmatic individuals facilitating recruitment and survival of eosinophils in the apical side of the bronchial epithelial barrier. The clinical relevance of this process is that eosinophilic airway inflammation has been linked to increased asthma severity [33]. Moreover, CCL11/eotaxin-1 levels in bronchoalveolar lavage fluid (BALF), exhaled breath condensate (EBC) and sputum are being investigated as potential biomarkers for the asthmatic condition [45]. Based on our current findings it is possible that a combination of TSLP/CCL11/eotaxin-1 can serve as a better marker of the TSLP-driven eosinophilic airway pro-asthmatic responses, and perhaps it might provide useful information for risk stratification and evaluation of treatment strategies for viral-induced asthma exacerbations.

In summary, this study is the first to identify intrinsic differences in the directional secretion of TSLP and CCL11/eotaxin-1 between normal and asthmatic differentiated airway epithelium. It is also the first to evaluate TSLP/CCL11/eotaxin-1 responses in asthmatic airway smooth muscle, a sub-epithelial tissue implicated in the pathologic bronchoconstrictive response seen in the asthmatics during viral respiratory illnesses. In addition, we provide new evidence of the in parallel airway secretion of TSLP/CCL11/eotaxin-1 during rhinovirus-induced asthma exacerbations in children. Our findings set the stage for future mechanistic studies to define the role of TSLP/CCL11/eotaxin-1 in virally mediated Th2 immune responses, as well as potential therapeutic studies designed to detect and attenuate virally induced changes in the bronchial epithelium and airway smooth muscle of asthmatic individuals.
